# Influence of state-of-the-art laboratory techniques on the phenotyping of women with polycystic ovary syndrome in the clinical setting

**DOI:** 10.1007/s40618-024-02416-0

**Published:** 2024-06-24

**Authors:** M. Luque-Ramírez, M. Á. Martínez-García, M. Insenser, E. Fernández-Durán, A. Quintero-Tobar, T. Fiers, J-M. Kaufman, A. M. García-Cano, M. Rosillo Coronado, L. Nattero-Chávez, H. F. Escobar-Morreale

**Affiliations:** 1 Diabetes, Obesity, and Human Reproduction Research Group, Centro de Investigación Biomédica en Red de Diabetes y Enfermedades Metabólicas Asociadas (CIBERDEM) from Instituto de Salud Carlos III, Instituto Ramón y Cajal de Investigación Sanitaria (IRYCIS) & Universidad de Alcalá, Madrid, Spain; 2 Department of Endocrinology and Nutrition, Hospital Universitario Ramón y Cajal, Carretera de Colmenar Viejo, KM 9.1, 28034 Madrid, Spain; 3 Laboratory for Hormonology, Department of Endocrinology, Ghent University Hospital, 9000 Ghent, Belgium; 4 Department of Biochemistry, Hospital Universitario Ramón y Cajal, Madrid, Spain

**Keywords:** Androgens, Diagnosis of endocrine disease, Reproductive endocrinology Ovary

## Abstract

**Purpose:**

Evidence-based guidelines for the management of polycystic ovary syndrome (PCOS) recommend clinical laboratories use liquid chromatography-tandem mass spectrometry (LC–MS/MS) for diagnosing biochemical hyperandrogenism. However, automated immunoassays are still mostly used in routine laboratories worldwide. Another hurdle for PCOS phenotyping in the clinical setting is ultrasound assessment of polycystic ovarian morphology. We address the impact of using state-of-the-art (LC–MS/MS) and of an anti-müllerian hormone (AMH) assay on the diagnosis of PCOS in routine practice.

**Methods:**

In a cross-sectional study, we included 359 premenopausal women consecutively evaluated because of symptoms of functional androgen excess or hyperandrogenemia, and finally diagnosed with PCOS. Patients were submitted to routine phenotyping based on serum androgen measurements by immunoassays and an ovarian ultrasound when necessary. Samples of all patients were also assayed by LC–MS/MS for hyperandrogenemia and for circulating AMH.

**Results:**

The observed agreement between immunoassays and LC–MS/MS in identifying hyperandrogenemia was poor [78.0%; k(95%CI): 0.366 (0.283;0.449)]. The observed agreement between ultrasound and increased AMH was 27.3% [(95%CI): 0.060 (0.005; 0.115)]. Using LC–MS/MS changed PCOS phenotypes in 60(15.8%) patients. Fifty-two (18.3%) individuals with hyperandrogenemia by routine immunoassays no longer presented with androgen excess by LC–MS/MS. Overall diagnostic agreement between routine assessment using immunoassays and ultrasound and that derived from LC–MS/MS and the addition of AMH to US was moderate [weighted κ (linear weights): 0.512 (0.416;0.608)].

**Conclusions:**

Immunoassays used in routine practice are unacceptably inaccurate for phenotyping women with PCOS. Our data cast some doubts upon the interchangeability of serum AMH and ultrasound examination for the diagnosis of PCOS.

**Supplementary Information:**

The online version contains supplementary material available at 10.1007/s40618-024-02416-0.

## Introduction

Current evidence-based practice guideline for the management of polycystic ovary syndrome (PCOS) recommends that clinical laboratories use validated liquid chromatography–tandem mass spectrometry (LC–MS/MS) assays for diagnosing biochemical hyperandrogenism in patients with PCOS [1]. Whilst automatized immunochemiluminescence (ICLA) or unextracted direct radioimmunoassays (RIA) are reliable enough to measure sex steroids such as dehydroepiandrosterone-sulfate (DHEAS), that circulate in μM concentrations [2], their diagnostic accuracy is poor at the very low nM circulating concentrations that characterize testosterone (T) or androstenedione (A4) in women [3–5]. The inaccuracy and lack of sensitivity of high-throughput ICLA for measuring sex steroids was reported almost two decades ago [6]. Nowadays, however, LC–MS/MS has not replaced ICLA in most routine clinical laboratories [7], despite the more favorable performance-to-cost ratio of the former.

In the clinical setting, a misdiagnosis of hyperandrogenemia derived from using ICLA, may lead to an inappropriate management in a significant proportion of women. Hyperandrogenemia identifies a subset of patients in whom metabolic risk is higher than that of their normoandrogenic counterparts [8]. On the other hand, a falsely elevated androgen concentration might submit affected women to harmful consequences such as the negative emotional impact of a lifetime diagnosis, prolonged follow-ups, or even unnecessary drug exposition [9].

In routine clinical practice, ultrasound (US) assessment of polycystic ovarian morphology (PCOM) represents another hurdle for PCOS phenotyping. Some US operators may lack the extensive training needed for the accurate assessment of ovarian volume and follicle number per ovary and, as a result, its diagnosis may become somewhat subjective. Moreover, the adequate assessment of PCOM constitutes a time-demanding exploration, the vaginal approach is not always feasible or desirable to the patient, and/or the imaging quality is insufficient for follicle counting [10]. To overcome these limitations, latest evidence-based guidelines recommend, with moderate quality of evidence, using also circulating anti-müllerian hormone (AMH) concentrations as a valid surrogate marker of PCOM in adults [1, 11].

We hypothesized that i) a more precise diagnosis of hyperandrogenemia would rule out the falsely elevated androgens that resulted from inaccurate immunoassays, avoiding overdiagnoses of PCOS; ii) the use of serum AMH concentrations for defining PCOM would increase the proportion of women fulfilling such diagnostic criteria. Hence, we here aimed to: i) compare, in a large series of women, the performances of state-of-the-art LC–MS/MS assays for hyperandrogenemia and of AMH assays for PCOM with those of routine immunoassays and US examinations conducted earlier; and ii) study how the implementation of these techniques would impact the distribution of PCOS phenotypes.

## Material and methods

### Subjects

We used serum samples and clinical data from 359 consecutive premenopausal women referred to our Reproductive Endocrinology clinic from 2006 to 2022 because of symptoms of functional androgen excess or hyperandrogenemia. To be included, a diagnosis of PCOS required the presence of at least two out of three criteria: clinical and/or biochemical androgen excess, ovulatory dysfunction (OD), and PCOM, and the woman had to allow us to include their database for research studies by signing an informed consent (see Ethical approval). We systematically excluded women with other etiologies of hyperandrogenism or ovulatory dysfunction [1, 12].

We defined clinical hyperandrogenism as hirsutism by a modified Ferriman-Gallwey score ≥ 8. In the clinical setting, immunoassays were used to estimate biochemical hyperandrogenism using local in-house cut-offs for each assay derived from a sample of non-hyperandrogenic premenopausal female volunteers presenting with regular menses (Table 1) [13, 14].

**Table 1:** Circulating androgen measurements by LC–MS/MS and serum anti-müllerian hormone assayed by the Elecsys ICLA immunoassay in a local non-hyperandrogenic control population

	Mean	Median	SD	Minimum	Maximum	95th percentile
Total testosterone, nM						
LC–MS/MS	1.0	1.0	0.3	0.4	1.9	1.6
ICLA	0.7	0.7	0.1	0.3	1.1	1.0
RIA	0.9	0.9	0.3	0.4	1.8	1.5
Calculated free testosterone, pM						
LC–MS/MS	13.9	13.9	5.2	2.8	31.9	23.2
ICLA	13.9	13.9	6.9	3.5	31.2	27.7
RIA	20.8	20.8	10.4	3.5	41.6	34.7
Androstenedione, nM						
LC–MS/MS	4.5	4.3	1.5	1.2	8.0	7.4
ICLA	8.7	8.5	2.9	2.8	17.5	13.8
Anti-müllerian hormone, pM	22.4	19.1	15.4	1.0	63.3	55.9

Control population for setting LC–MS/MS normative values (n = 91) was comprised of female volunteers presenting with regular menses derived from the hospital's staff and overweight or obese women seeking advice solely for weight loss at our Department. Their age, body mass index, and waist circumference (mean ± SD) were 29 ± 6 yrs, 26.6 ± 7.6 kg/m^2^, and 82 ± 17 cm, respectively. Twenty-nine of them had obesity as defined by a body mass index ≥ 30 kg/m^2^. Control population features for defining normative values of RIA and ICLA assays is described in detail elsewhere [14]. Free testosterone was calculated by the Vermeulen’s formula using a default albumin level of 43 g/L. Mean sex hormone-binding globulin values were 54 ± 25 nM

*ICLA* automatized immunochemiluminescence, *LC-MS/MS* liquid chromatography–tandem mass spectrometry, *RIA* unextracted direct radioimmunoassay

World Health Organization (WHO) group II OD (https://www.ncbi.nlm.nih.gov/books/NBK327781/) was defined by the presence of more than six cycles longer than 36 days in the previous year, absence of menstruation for three consecutive months, or by luteal phase progesterone concentrations below 17 μM (4 ng/mL) in women with regular menses.

Ovarian US examination was conducted in those women who presented with only hyperandrogenism or OD. On the contrary, in women meeting both criteria US was not systematically performed because they had already met a PCOS diagnosis, yet many of these women also received an US examination as a part of their Gynecology consultation [1]. PCOM was evaluated by gynecologists according to routine clinical practice based on the European Society of Human Reproduction and Embryology/American Society for Reproductive Medicine 2003 criteria [15]. Rotterdam criteria defined PCOM by the presence of ≥ 12 follicles in each ovary measuring 2–9 mm in diameter, and/or increased ovarian volume (10 mL) estimated both in longitudinal and antero-posterior cross-sections of the ovaries. According to current evidence-based definition for PCOM [1], an ovarian volume ≥ 10 ml or follicle number per section ≥ 10 in at least one ovary in adults can be considered the threshold for PCOM when older technology or image quality is insufficient to allow for an accurate assessment of follicle counts throughout the entire ovary; hence, those examinations would fulfill 2023 criteria also for clinical practice. None of these women had received treatment with oral contraceptives, antiandrogens, or insulin sensitizers during at least 6 months before sampling.

### Metabolic phenotyping

Two trained investigators (M.L.-R. & H.F.E.-M.) were responsible for clinical, anthropometric, and physical evaluations, including the above-mentioned hirsutism score, body mass index (BMI), and waist circumference using the National Health Examination Survey method [16]. Office blood pressure was determined as the mean of two manual sphygmomanometer readings at sitting position. The composite insulin sensitivity index was calculated from the circulating glucose and insulin concentrations during a standard oral glucose tolerance test (oGTT) [17]. Abnormal glucose tolerance (prediabetes and type 2 diabetes mellitus) was defined from circulating glucose concentrations at 0 and 120 min during oGTT [18].

### Sampling

Basal blood samples for sex steroid profiles and serum AMH were obtained after 12-h overnight fasting, between days 3 and 9 of a spontaneous or progestin withdrawal-induced menstrual bleeding, or at random after excluding pregnancy in amenorrheic patients. Then, a 75-g oGTT was performed, and samples were obtained at 0, 30, 60, 90, and 120 min. Samples were immediately centrifuged, and serum and plasma for storing were aliquoted, coded, and frozen at − 80 °C until thawed for analysis.

### Assays

The technical specifications of sex steroids and serum AMH assays are detailed in Supplementary Information. For LC–MS/MS measurements and AMH assay, biochemical hyperandrogenism and upper limit of normality (ULN) were defined by the presence of values > 95th percentile (P95) of a sample of non-hyperandrogenic premenopausal female volunteers presenting with regular menses, composed of hospital's staff and overweight or obese women seeking advice solely for weight loss at our Department (Table 1). Those control women were similar in terms of BMI and age to the study population of hyperandrogenic women. None of these controls had a history of infertility, oophorectomy, or hysterectomy nor had received treatment with hormonal contraceptives, antiandrogens, or insulin sensitizers for at least 6 months before sampling. Of note, this population of control women derived from a similar sample of non-hyperandrogenic individuals than that previously used in establishing local in-house normality values for routine androgen assays [13, 14]. In the subset of women in whom sonography exploration was available, we also determined the optimal AMH threshold value in identifying PCOM by means of the Youden’s J statistics.

All but the LC–MS/MS and serum AMH assays were run at the time of subjects’ recruitment as described above. Both the external and local laboratories were blinded to women's features and patient or control status.

### Statistical analysis

Data are shown as mean ± standard deviation or 95% confidence intervals (95%CI), and counts (percentage). For continuous variables, their normal distribution was assessed by the Kolmogorov–Smirnov test, and logarithmic transformation was applied to ensure normality if needed. First, we compared T and A4 levels as measured by routine immunoassays and LC–MS/MS, and their relationships with anthropometric, clinical, and metabolic variables, in the whole group of hyperandrogenic women. Second, we compared subgroups of hyperandrogenic women among them and with non-hyperandrogenic control women.

Continuous variables were compared by one-way ANOVA, Welch´s ANOVA, or univariate general linear models as a function of the homogeneity of variances and adjustment by covariates. Mean differences among three or more groups were analyzed by post hoc Tukey, Games-Howell, or Bonferroni methods. Categorical variables were analyzed by Fisher's exact, χ^2^ tests, or binary logistic regression models. Instead of using Passing-Bablok or Deming regressions, Spearman's correlation and linear regression were used to compare routine immunoassays with LC–MS/MS results since the latter is considered as the gold-standard method for sex steroid measurement. Consistency and absolute agreement among assays were addressed by their intra**-**class correlation coefficient using a two-factor and a random-effect model. Quantitative agreement was graphically assessed by Bland–Altman plots and the ratio method. Diagnostic agreement between routine phenotyping and those phenotypes derived from applying LC–MS/MS and AMH measurements was assessed by kappa (*κ*) and weighted *κ* coefficients [MedCalc Software Ltd. Inter-rater agreement. https://www.medcalc.org/calc/kappa.php (Version 22.014; accessed May 9, 2024)]. We performed other statistical analyses using IBM® SPSS® Statistics 23 (IBM España S.A., Madrid, Spain).

A *P* value < 0.05 was considered statistically significant.

## Results

### Different assays when diagnosing hyperandrogenism

The clinical variables, anthropometrics and sex steroid profiles of our study population are shown in Table 2. Routine total T was assayed by RIA and ICLA in 306 (85.2%) and 53 (14.8%) women, respectively (Supplementary Table S1). Total T and A4 concentrations assayed by routine methods showed a moderate positive correlation with those derived from LC–MS/MS, but the concordance among both techniques was poor-moderate in terms of total T, and virtually inexistent for A4 (Supplementary Information, Figure S1). Bland–Altman plots showed a tendency towards greater differences with increasing mean T and A4 values, especially with the former, and when those samples had been assayed by RIA. This total T overestimation by the routine immunoassays was confirmed using the ratio method.

**Table 2:** Clinical and anthropometric variables, sex steroids and metabolic profiles of women with PCOS

	N	Mean/Count	SD/%	Median	Interquartile range
Age, years	359	27	7	27	10
Body mass index, kg/m^2^	359	27.2	7.3	25.1	10.2
Waist circumference, cm	290	82	17	78	13
Waist-to-hip ratio	282	0.80	0.10	0.78	0.11
Obesity	359	122	34.0%	–	–
Systolic blood pressure, mmHg	347	119	14	120	17
Diastolic blood pressure, mmHg	347	75	10	75	13
Hirsutism	356	142	39.6%	–	–
Routine immunoassay total testosterone, nM	359	2.0	1.0	1.8	1.2
LC–MS/MS total testosterone, nM	359	1.2	0.6	1.1	0.7
Sex hormone–binding globulin, nM	359	44	27	37	32
Routine immunoassay cFT, pM	359	39.7	36.5	21.7	26.5
LC–MS/MS cFT, pM	359	23.5	12.3	20.8	13.1
Routine immunoassay androstenedione, nM	356	12.5	5.4	12.0	7.0
LC–MS/MS androstenedione, nM	359	6.49	2.62	6.14	3.18
Dehydroepiandrosterone–sulphate, μM	359	6.5	3.2	5.9	4.1
Hyperandrogenemia (Routine immunoassays)	359	284	79.1%	–	–
Hyperandrogenemia (LC–MS/MS)	359	214	59.6%	–	–
Ovulatory dysfunction	359	335	93.3%	–	–
PCOM by ultrasound	209	178	85.2%	–	–
Anti–müllerian hormone, pM	359	47.3	34.5	39.2	37.5
Increased anti-müllerian hormone (> 55.9 pM)	357	109	30.4%	–	–
Fasting plasma glucose, mM	353	4.9	0.6	4.8	0.7
120-min plasma glucose, mM	350	6.5	2.1	6.2	2.0
AUC _oGTT glucose_, mM*120 min	349	249	177	232	214
Insulin sensitivity index	346	6.2	4.4	5.3	5.6
Total cholesterol, mM	359	4.6	0.9	4.5	1.1
HDL-cholesterol, mM	351	1.4	0.3	1.3	0.4
LDL-cholesterol, mM	351	2.7	0.8	2.7	0.9
Triglycerides, mM	359	1.0	0.6	0.8	0.5

*AUC* area under the curve, *cFT* calculated free testosterone, *LC–MS/MS,* liquid chromatography-tandem mass, *oGTT* standard oral glucose tolerance test, *PCOM* polycystic ovarian morphology

### Impact on PCOS phenotyping of LC–MS/MS and serum anti-müllerian hormone

Table 3 shows the counts and percentages of hyperandrogenemia as a function of the assay used for measurement of serum androgen concentrations. The agreement between immunoassays and LC–MS/MS in identifying those patients with or without hyperandrogenemia was poor, regardless of the circulating androgen being analyzed; this occurred mostly because approximately 20% women showing normal LC–MS/MS values had shown hyperandrogenemia when using routine immunoassays.

**Table 3:** Presence of hyperandrogenemia as a function of sex steroid assay in women with PCOS

	Immunoassay & LC–MS/MS	Only in immunoassay	Only in LC–MS/MS	Normal androgens	κ coefficient (95%CI)
Total testosterone					
RIA or ICLA	104 (29.0)	145 (40.4)	7 (1.9)	103 (28.7)	0.262 (0.195; 0.330)
RIA	89 (29.9)	127 (41.5)	5 (1.6)	85 (27.8)	0.256 (0.185; 0.326)
ICLA	15 (28.3)	18 (34.0)	2 (3.8)	18 (34.0)	0.306 (0.103; 0.509)
Free calculated testosterone	130 (36.2)	74 (20.6)	19 (5.3)	136 (37.9)	0.494 (0.409; 0.578)
Total or free testosterone	165 (46.0)	104 (29.0)	13 (3.6)	77 (21.4)	0.351 (0.268; 0.434)
Androstenedione	67 (18.8)	39 (11.0)	38 (10.7)	212 (59.6)	0.481 (0.382; 0.581)
Total or free testosterone and/or androstenedione	175 (48.7)	102 (28.4)	10 (2.8)	72 (20.1)	0.366 (0.283; 0.449)
All androgens including DHEAS	205 (57.1)	79 (22.0)	9 (2.5)	66 (18.4)	0.448 (0.359; 0.537)

Hyperandrogenic women were categorized as follows: women showing biochemical hyperandrogenism by both our routine immunoassays (ICLA or RIA) and by LC–MS/MS; women showing hyperandrogenemia only by one of those assays (by routine immunoassays only or by LC–MS/MS only); and women with normal circulating androgens by both assays. Data are counts (percentages) within each subgroup. The diagnostic agreement between assays was analyzed by the κ coefficient (95% confidence interval). Measuring circulating A4 by LC–MS/MS only increased the number of women with a diagnosis of hyperandrogenemia by 3.9 percentage points compared with those diagnosed based on total or calculated free testosterone measurements. On the contrary, 20.2% women showed an elevated circulating DHEAS as the only marker of hyperandrogenemia

*DHEAS* dehydroepiandrosterone-sulphate, *ICLA* immunochemiluminescence assay, *LC–MS/MS* liquid chromatography-tandem massliquid chromatography-tandem mass, *RIA* radioimmunoassay

One hundred-seventy-eight out of 209 women who received sonographic evaluation presented with PCOM (85.2%) (Table 2). Using the P95 of AMH in our control group of non-hyperandrogenic women as cut-off value [55.9 pM (7.8 ng/mL)], 109 (30.5%) out of 357 patients showed increased circulating AMH concentrations (two samples were missing). In those women with US assessment, the diagnostic agreement between US-PCOM and AMH was only 27.3% [κ: 0.060 (0.005; 0.115)]. After performing a receiver-operating characteristic (ROC) curve analysis (Supplementary Information, Figure S2), AMH showed a poor diagnostic performance in diagnosing PCOM. According to the Youden’s index, the optimal AMH threshold value would be 16.3 pM (2.3 ng/mL), which showed 0.92 sensitivity for diagnosing ultrasound PCOM, but only 0.36 specificity. To control for the effect of inter-observer variability and diverse US equipment, we also restricted these analyses to those subjects evaluated throughout the last 5 years of the inclusion period─namely, from 2017 to 2022─in which the same gynecologists conducted those explorations. In these subjects (*n* = 116), the diagnostic agreement between US-PCOM and AMH was also poor: 43.1% [κ: 0.115 (0.039; 0.191)].

**Fig. 1:**
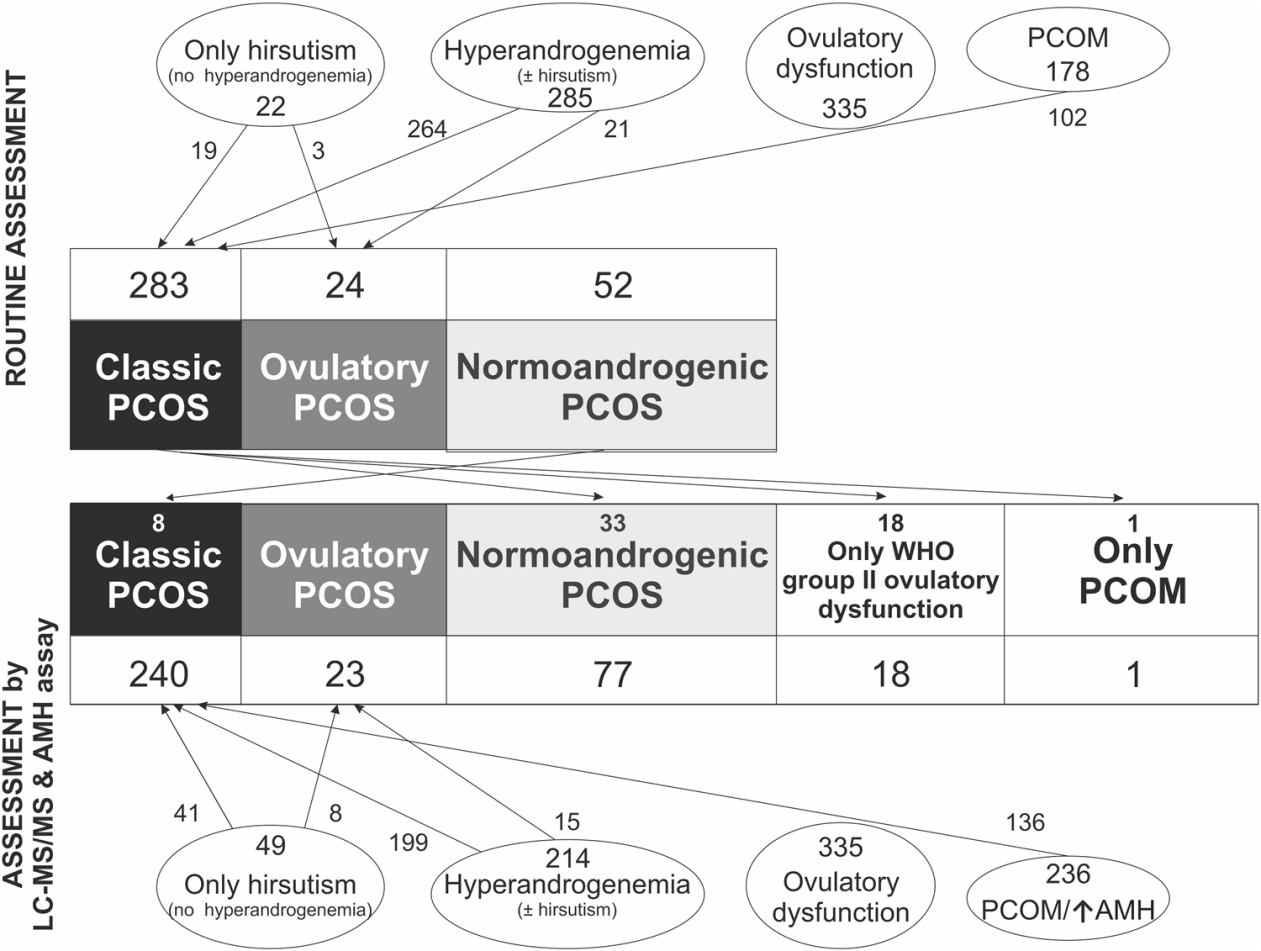
PCOS phenotypes identified as a function of each individual diagnostic criteria by routine methods of androgen assay and PCOM examination (upper panel) compared to gold standard LC–MS/MS for diagnosing hyperandrogenemia and serum AMH support (lower panel). The numbers into the white boxes indicate how many subjects are included in each PCOS phenotype. The arrows between upper and lower boxes denotes women who changed their PCOS phenotype after the study. The small number above final phenotypes indicate how many individuals updated their phenotype

Figure 1 shows PCOS phenotypes after initial routine assessment using androgen immunoassays and US, and their re-classification after applying LC–MS/MS and adding AMH plus US to define hyperandrogenemia and PCOM, respectively. Fifty-two (18.3%) out of 285 individuals with hyperandrogenemia according to routine immunoassays (264 patients with classic PCOS and 21 women with ovulatory PCOS) no longer presented with androgen excess by LC–MS/MS. These 52 women were now diagnosed as non-hyperandrogenic PCOS in 26 cases (50.0%), group II WHO OD in 25 cases (48.1%), and isolated PCOM in the remaining case, even though only six out of those 25 women in the group II WHO OD had initially received an ovarian US examination. On the contrary, eight (15.4%) of the 52 women initially diagnosed with non-hyperandrogenic PCOS by routine methods were re-classified into the classic PCOS phenotype after using LC–MS/MS (Fig. 1). Overall, 60 out of 359 (16.7%) patients changed their PCOS phenotype after applying LC–MS/MS. The diagnostic agreement among PCOS phenotypes resulting from the application of routine immunoassays or from the use of LC–MS/MS was moderate [weighted κ (linear weights): 0.500 (0.404; 0.596)].

When we added an elevated serum AMH concentration as a surrogate marker for PCOM to the ovarian US conducted initially, seven out of those 25 patients with group WHO II OD now fulfilled diagnostic criteria of non-hyperandrogenic PCOS. However, an elevated AMH concentration as a surrogate marker of PCOM would have only identified three out of 23 women diagnosed with ovulatory PCOS when using ovarian US, and only 25 out of 77 women with non-hyperandrogenic PCOS according to US showed increased AMH values. In other words, 20 (87.0%) patients initially diagnosed with ovulatory PCOS, and 52 (67.5%) of those diagnosed with non-hyperandrogenic PCOS, had PCOM by US but a serum AMH ≤ 55.9 pM (7.8 ng/mL). Overall diagnostic agreement between routine assessment using immunoassays and US and that derived from LC–MS/MS and the addition of AMH to US was moderate [weighted κ (linear weights): 0.512 (0.416; 0.608)]. If we do not take into account its poor specificity, using the cut-off derived from Youden’s statistics would have been classified as normoandrogenic PCOS, 20 out of those 25 women presenting with only OD. Nonetheless, overall diagnostic agreement between routine assessments and LC–MS/MS and the addition of AMH to US was not improved [weighted κ (linear weights): 0.536 (0.441; 0.632)]. Similarly, when we tested the AMH cutoff based on a previous study using the Elecsys AMH Plus immunoassay [3.2 ng/mL (23 pM)] [19], 18 out of that group of 25 women were classified as normoandrogenic PCOS.

### Effect of changes in the phenotyping of patients with PCOS on their metabolic profiles

The only subgroup of patients in whom metabolic indices were altered when compared with controls was that comprised of women showing hyperandrogenemia by LC–MS/MS, regardless of immunoassays; these patients presented with reduced  insulin sensitivity index and HDL-cholesterol, and increased diastolic BP values and triglycerides concentrations, and were more likely to have an abnormal glucose metabolism, namely prediabetes or diabetes mellitus (Table 4). Moreover, their insulin sensitivity index was lower, and their triglyceride concentrations and serum AMH concentrations were higher, than the subgroup of women showing hyperandrogenemia only by immunoassays.

**Table 4:** Anthropometrics, clinical and metabolic features when grouping patients as a function of the method used to diagnose hyperandrogenemia

	Patients				Control women (*n* = 91)
	Isolated hirsutism	Hyperandrogenemia by LC–MS/MS regardless of immunoassays	Hyperandrogenemia only by immunoassays	No evidence of androgen excess	
	(*n* = 44)	(*n* = 214)	(*n* = 79)	(*n* = 22)	
Age, years^a,b^	27 ± 8	26 ± 6^*^	28 ± 7	29 ± 5	29 ± 6
Body mass index, kg/m^2^	25.5 ± 7.2	27.5 ± 7.7	26.9 ± 6.3	27.2 ± 7.1	26.6 ± 7.6
Obesity	6 (27)	74 (35)	28 (36)	13 (30)	29 (32)
Waist circumference, cm	81 ± 20	82 ± 18	81 ± 14	83 ± 18	82 ± 17
Waist-to-hip ratio	0.79 ± 0.11	0.81 ± 0.12	0.79 ± 0.06	0.78 ± 0.08	0.79 ± 0.09
Systolic blood pressure, mmHg	118 ± 13	118 ± 13	119 ± 13	122 ± 11	113 ± 12
Diastolic blood pressure, mmHg^a^	74 ± 10	75 ± 10^*^	75 ± 9	77 ± 10	73 ± 10
Fasting glucose, mM	5.0 ± 0.7	5.0 ± 0.6	4.8 ± 0.4	4.9 ± 0.4	4.9 ± 0.5
120 min-oGTT glucose, mM	5.9 ± 1.3	6.6 ± 1.9	6.6 ± 2.8	5.9 ± 1.4	6.3 ± 1.5
AUC _oGTT glucose_, mM*120 min	209 ± 134	256 ± 154	276 ± 236	186 ± 166	246 ± 121
Insulin sensitivity index^a,b,c^	7.0 ± 4.1	5.5 ± 3.9^*^	6.9 ± 5.5	8.0 ± 4.3	7.2 ± 3.6
Abnormal glucose tolerance	5 (24)	63 (30)	17 (22)	8 (18)	18 (20)
Total cholesterol, mM	4.7 ± 0.8	4.6 ± 0.8	4.4 ± 0.9	4.5 ± 0.8	4.6 ± 0.9
HDL, cholesterol, mM^a^	1.3 ± 0.2	1.4 ± 0.3^*^	1.4 ± 0.3	1.5 ± 0.4	1.5 ± 0.4
LDL-cholesterol, mM	2.9 ± 0.8	2.8 ± 0.7	2.6 ± 0.9	2.6 ± 0.6	2.8 ± 0.7
Triglycerides, mM^a,b,c^	1.1 ± 0.7	1.1 ± 0.6^*^	0.9 ± 0.5	0.8 ± 0.4	0.9 ± 0.5
Anti-müllerian hormone, pM^a,c^	34.5 ± 17.9^*^	53.4 ± 39.1^*^	37.9 ± 26.3^*^	40.3 ± 21.2^*^	22.3 ± 15

Data are means ± SD or counts (%). Continuous variables were compared among groups by univariate ANOVA, Welch-ANOVA or univariate-GLM analysis adjusted by age if necessary, and followed by Tukey’s, Games-Howell’s or Bonferroni’s post-hoc analysis, respectively. Dichotomous variables were compared by binary logistic regression analysis adjusting by age

*AUC* area under the curve, *oGTT* oral glucose tolerance test

* *P* < 0.05 vs. control group

^a^ *P* < 0.05 between all subgroups

^b^ *P* < 0.05 for post-hoc comparisons between patients with hyperandrogenemia by LC–MS/MS *vs.* those with isolated hirsutism

^c^ *P* < 0.05 for post-hoc comparisons between patients with hyperandrogenemia by LC–MS/MS *vs.* those with hyperandrogenemia by only immunoassay

Table 5 shows anthropometrics, clinical and metabolic variables as a function of PCOM in 209 women in whom both ovarian US and AMH concentrations were available. Only 4 women had PCOM as defined by AMH in the presence of a normal ovarian US. Women with US-PCOM showed higher BMI values and indexes of abdominal adiposity compared with those in whom PCOM was established by AMH. Moreover, women with PCOM according to both US and AMH showed higher circulating total T and A4 concentrations than those only matching US criteria for PCOM.

**Table 5:** Anthropometrics, clinical and metabolic features as a function of PCOM status

	Patients with both US & serum AMH available data				Control women (n = 91)
	PCOM by US/↑AMH	Only PCOM by US	Only PCOM by ↑AMH	No PCOM	
	(*n* = 51)	(*n* = 127)	(*n* = 4)	(*n* = 27)	
Age, years	28 ± 6	27 ± 7	23 ± 8	27 ± 8	29 ± 6
Body mass index, kg/m^2^	27.8 ± 6.6	26.6 ± 6.5	23.0 ± 8.3	26.7 ± 8.2	26.6 ± 7.6
Obesity	16 (31.4)	48 (37.8)	0 (0)	10 (37.0)	29 (32)
Waist circumference, cm^a,b,c,d^	83 ± 15	83 ± 18	63 ± 2^*^	82 ± 18	82 ± 17
Waist-to-hip ratio^a,b^	0.81 ± 0.09	0.80 ± 0.12	0.66 ± 0.03^*^	0.77 ± 0.07	0.79 ± 0.09
Systolic blood pressure, mmHg^a^	119 ± 12	120 ± 14^*^	108 ± 12	121 ± 9^*^	113 ± 12
Diastolic blood pressure, mmHg	76 ± 9	75 ± 11	66 ± 4	75 ± 9	73 ± 10
Fasting glucose, mM	4.9 ± 0.5	5.0 ± 0.6	4.6 ± 0.6	4.9 ± 0.7	4.9 ± 0.5
120 min-oGTT glucose, mM	6.3 ± 1.6	6.4 ± 1.7	5.4 ± 0.5	6.9 ± 3.8	6.3 ± 1.5
AUC_oGTT glucose_, mM*120 min	221 ± 158	231 ± 155	194 ± 49	246 ± 121	246 ± 121
Insulin sensitivity index	7.2 ± 4.7	6.3 ± 5.1	8.5 ± 5.8	6.0 ± 3.3	7.2 ± 3.6
Abnormal glucose tolerance	10 (20)	34 (27.2)	0 (0)	7 (25.9)	18 (20)
Total cholesterol, mM	4.7 ± 0.8	4.5 ± 0.9	4.3 ± 0.6	4.4 ± 0.7	4.6 ± 0.9
HDL, cholesterol, mM	1.5 ± 0.4	1.3 ± 0.3	1.4 ± 0.2	1.3 ± 0.2	1.5 ± 0.4
LDL-cholesterol, mM	2.8 ± 0.7	2.7 ± 0.8	2.6 ± 0.5	2.5 ± 0.5	2.8 ± 0.7
Triglycerides, mM	1.0 ± 0.6	1.0 ± 0.6	0.7 ± 0.2	1.0 ± 0.6	0.9 ± 0.5
Anti-müllerian hormone, pM^a,c,d,e,f^	92.8 ± 39.3^*^	30.7 ± 12.9^*^	71.4 ± 14.3^*^	27.1 ± 15.7	22.3 ± 15.4
Total testosterone, nM^a,e,f^	1.6 ± 0.7^*^	1.2 ± 0.5^*^	1.7 ± 0.7	1.2 ± 0.4	0.8 ± 0.3
Sex hormone-binding globulin, nM^a^	55 ± 32	43 ± 26^*^	44 ± 18	39 ± 23^*^	54 ± 25
Calculated free testosterone, pM^a^	25.2 ± 16.7^*^	21.2 ± 11.4^*^	25.1 ± 6.3	19.3 ± 6.1^*^	14.0 ± 5.3
Androstenedione, nM^a,e^	7.3 ± 2.9^*^	5.7 ± 2.0^*^	7.2 ± 2.0	5.7 ± 2.0^*^	4.5 ± 1.5
Dehydroepiandrosterone-sulphate, μM	5.8 ± 3.2	6.2 ± 3.3	7.1 ± 2.8	6.4 ± 3.9	5.0 ± 2.4
PCOS phenotype					
Classic	28 (55)	56 (44)	4 (100)	21 (78)	–
Ovulatory	3 (6)	20 (16)	0 (0)	0 (0)	–
Normoandrogenic	20 (39)	50 (39)	0 (0)	0 (0)	–
No PCOS	0 (0)	1 (1)	0 (0)	6 (22)	–

Data are shown as means ± SD or counts (%). *Abbreviations,* ↑AMH, anti-müllerian hormone above the 95th percentile of the control group; PCOM, polycystic ovarian morphology; US, ultrasound. ^*^P < 0.05 vs. control group; ^a^*P* < 0.05 between all subgroups; ^b^*P* < 0.05 between patients with PCOM by US & ↑AMH *vs.* those only with PCOM by ↑AMH; ^c^*P* < 0.05 between patients with only PCOM by US *vs.* those only with PCOM by ↑AMH.; ^d^*P* < 0.05 between patients with only PCOM by ↑AMH *vs.* those with no PCOM; ^e^*P* < 0.05 between patients with PCOM by US & ↑AMH *vs.* those only with PCOM by US, ^f^*P* < 0.05 between patients with PCOM by US & ↑AMH *vs.* those with no PCOM

*AMH* anti-müllerian hormone, *AUC* area under the curve, *oGTT* oral glucose tolerance test, *PCOM* polycystic ovarian morphology

Those presenting with elevated AMH had reduced BMI and systolic BP, and increased insulin sensitivity index, circulating T and A4 concentrations, and HDL-cholesterol compared with their counterparts showing normal AMH. When the analysis was restricted to patients with ovulatory or non-hyperandrogenic phenotypes, those presenting with elevated AMH concentrations were leaner, had a higher insulin sensitivity index and circulating HDL-cholesterol, SHBG, total T and A4, but showed lower DHEAS concentrations than women with normal AMH levels (Supplementary Information, Tables S2 & S3).

**Fig. 2:**
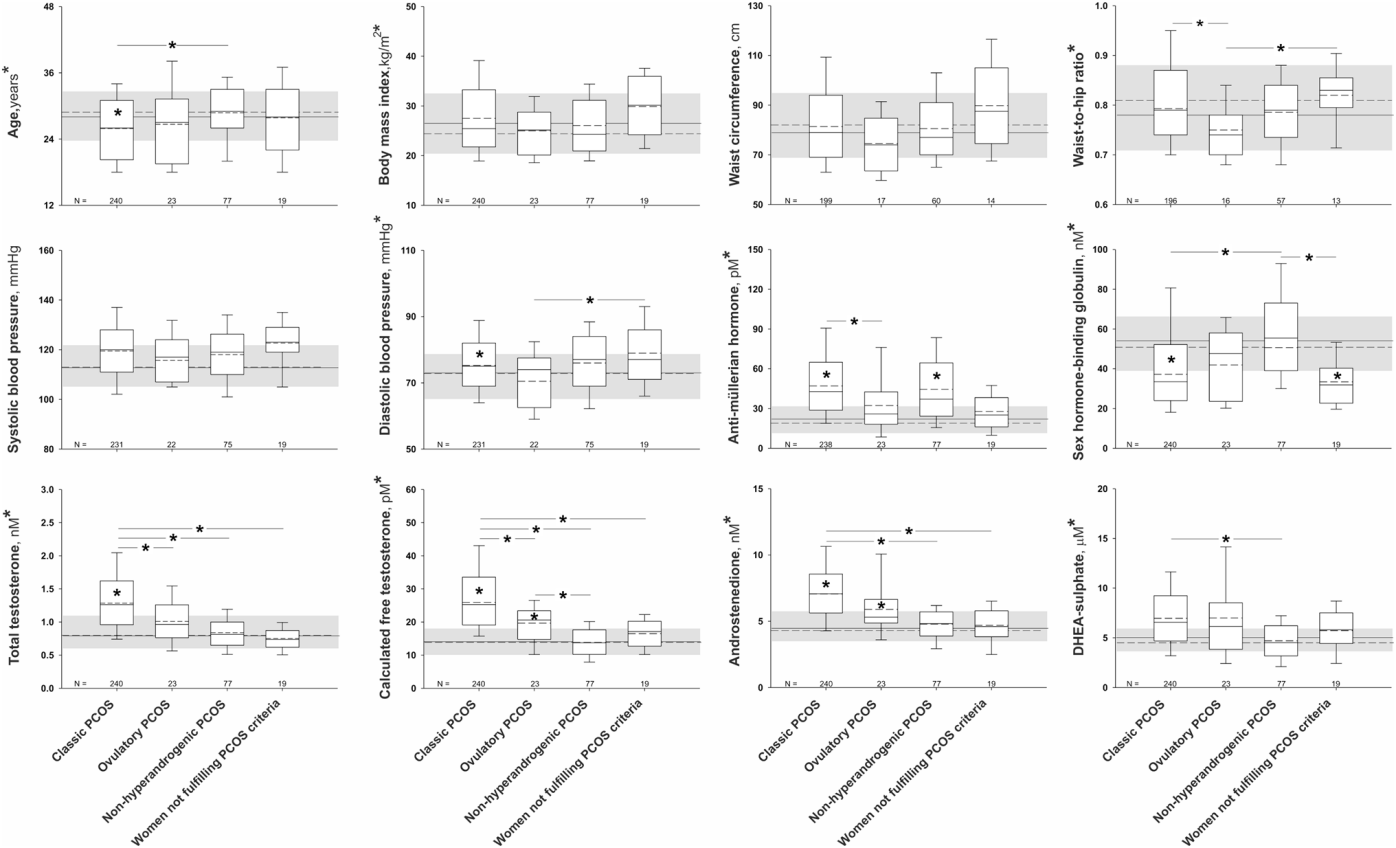
Anthropometric, clinical variables, circulating androgens, and anti-müllerian hormone levels according to revised PCOS phenotypes. The box indicates the 25th and 75th percentiles, the solid and short dashed lines within the boxes mark the median and mean, respectively. Whiskers below and above the box indicate the 10th and 90th percentiles. The shaded areas, solid and short dashed lines behind the boxes represent the interquartile range, median and mean, respectively, of our control population. * (y-axis) significant differences for the comparisons among all subgroups of patients including the control group of women. * (within the boxes) significant differences between that PCOS phenotype and control women. * (above the boxes) significant differences between those PCOS phenotypes

**Fig. 3:**
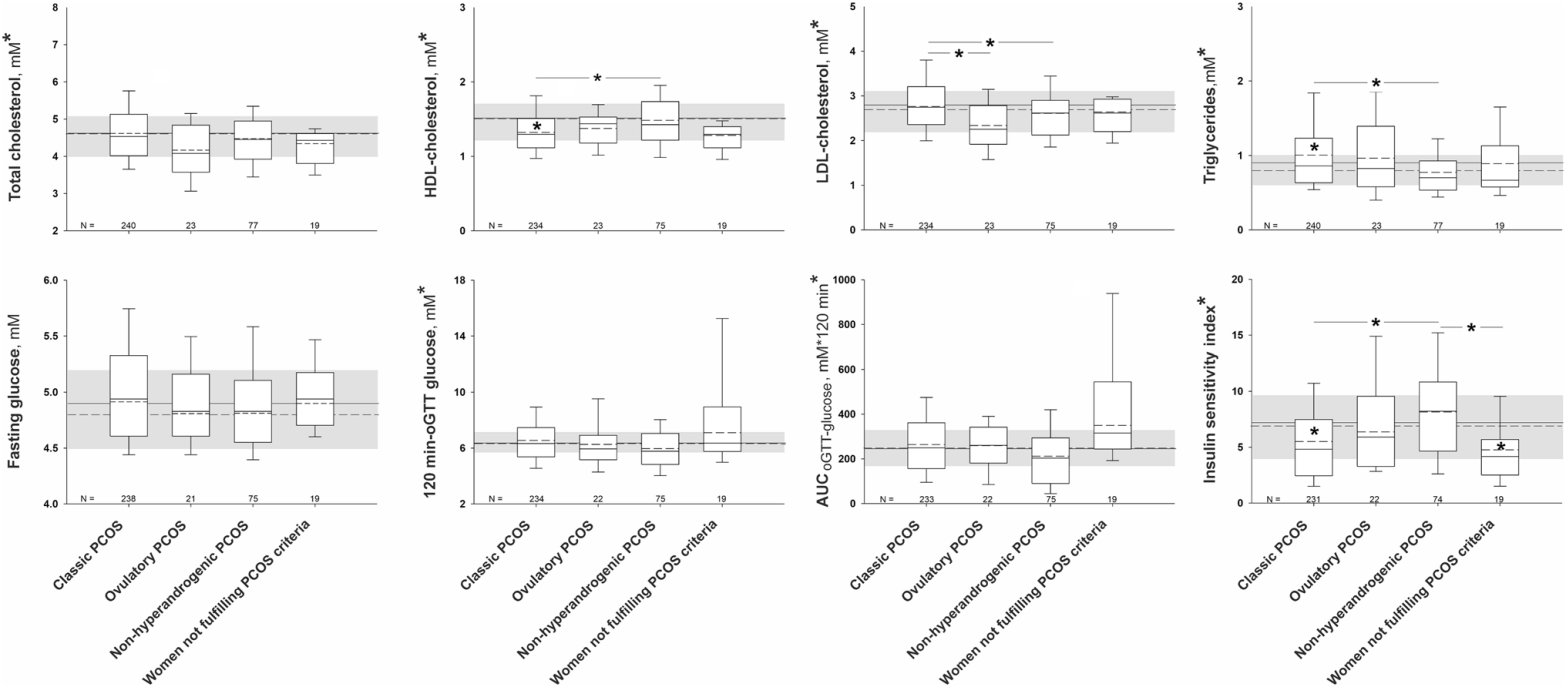
Lipid profile and glucose metabolism parameters according to revised PCOS phenotypes. The box indicates the 25th and 75th percentiles, the solid and short dashed lines within the boxes mark the median and mean, respectively. Whiskers below and above the box indicate the 10th and 90th percentiles. The shaded areas, solid and short dashed lines behind the boxes represent the interquartile range, median and mean, respectively, of our control population. * (y-axis) significant differences for the comparisons among all subgroups of patients including the control group of women. * (within the boxes) significant differences between that PCOS phenotype and control women. * (above the boxes) significant differences between those PCOS phenotypes

The re-classification of PCOS phenotypes resulting from the application of LC–MS/MS and addition to serum AMH concentrations to ovarian US confirmed the association of hyperandrogenic phenotypes with metabolic dysfunction. Despite all subgroups of women showing a similar BMI, only patients with the classic PCOS phenotype presented with higher mean diastolic BP, circulating triglycerides, and lower mean HDL-cholesterol concentrations compared with the control group (Figs. 2 & 3). Mean LDL-cholesterol was also higher in patients with classic PCOS than in ovulatory and non-hyperandrogenic phenotypes. This latter non-hyperandrogenic phenotype showed a better lipid profile also for HDL-cholesterol and triglycerides compared with classic PCOS.

Regarding carbohydrate metabolism, patients with classic PCOS had reduced insulin sensitivity index compared with control women and those with non-hyperandrogenic PCOS (Fig. 3). Furthermore, only women with the classic PCOS phenotype were more likely to have prediabetes or diabetes mellitus than control women [OR: 1.86 (1.02; 3.40); *P* = 0.043; Supplementary Information, Figure S3]. Of note, those women not fulfilling PCOS criteria after applying LC–MS/MS and serum AMH assessments –almost all women with group II WHO OD– showed an insulin sensitivity index that was similar to that of women with classic PCOS and was lower than that of control women (Fig. 3). Five of them (26.3%) showed abnormal glucose tolerance –including type 2 diabetes in 3 women– that was accompanied by obesity in four cases.

## Discussion

Automated ICLA is still mostly used in routine laboratories worldwide [7, 20, 21], despite the repeated recommendations to use LC–MS/MS for the measurement of total T in women made by most international scientific societies during the past 15 years [1, 22, 23]. Although standardization efforts may have enhanced analytical accuracy and precision for T immunoassays, these improvements still do not permit an accurate measurement at the very low concentrations that characterize non-hyperandrogenic women or patients with PCOS [20]. In agreement, the application of a validated LC–MS/MS T and A4 assay to serum samples from our clinically well-characterized population of hyperandrogenic women and controls, revealed that over 20% of women diagnosed with hyperandrogenemia by immunoassay had normal androgen concentrations. As a result, the diagnosis of 17% of the patients changed from hyperandrogenic to non-hyperandrogenic phenotypes of PCOS, and more importantly, in as many as 5% of the women previously diagnosed with PCOS such a diagnosis could not be confirmed any longer. Considering the very large prevalence of PCOS reported worldwide, these apparently small figures become of utmost importance in terms of economic burden, besides carrying an unnecessary psycho-emotional stigma for women incorrectly diagnosed with this condition.

Our data partially agree with previous observations comparing routine phenotyping by ICLA against LC–MS/MS in a population of 204 Italian women with PCOS [3]. In this Italian series, 15.7% women diagnosed with ICLA-hyperandrogenemia had normal circulating androgen levels by LC–MS/MS. Conversely, LC–MS/MS unmasked hyperandrogenemia in another 13.7% classified as normoandrogenic by immunoassays [3]. As a result, 12.2% of patients were reassigned to a different PCOS phenotype, with most of them moving from the normoandrogenic to the classic phenotype.

Both reports confirm the poor-performance of immunoassays when compared with the gold-standard LC–MS/MS technique, although some differences among studies merit further explanations. The use of LC–MS/MS for the diagnosis of hyperandrogenemia and serum AMH for supporting PCOM definition in our series changed the PCOS phenotype of 16.8% women but, unlike the findings of the Italian study, most of our patients were reassigned from the classic phenotype to the normoandrogenic one. This apparent discrepancy may rely on: i) we used two different immunoassays throughout the recruitment period previously compared in terms of performance for phenotyping women with PCOS [14]. Our unextracted direct RIA found hyperandrogenemia in 17.2% more women than the ICLA did. However, this difference had a small impact on PCOS phenotyping when clinical hyperandrogenism was also considered, reducing the 17.2% figure of changes in the phenotype to merely 3.2% [14]; ii) the ULN for testosterone based on the P95 of our control group of women was slightly higher than that reported in the Italian population, possibly because we matched our control population for BMI with the patients, and the Italian controls were normal-weight women; and iii) most participants (90%) in the Italian study presented with PCOM according to US, whereas we performed an ovarian US in only 58% of our patients, even though 85% of them showed PCOM.

At this point, was serum AMH measurement helpful in supporting PCOS phenotyping in our population? There is no doubt that serum AMH measurement would simplify PCOS phenotyping in the routine practice, avoiding explorations that may not only be considered unpleasant by women, but may also lead to misdiagnoses derived from untrained observers or obsolete point-of-care US equipment. Furthermore, serum AMH may play a role in clinical counseling regarding assisted reproductive therapy outcomes among women with PCOS undergoing fertility treatment [24]. Nevertheless, if an elevated serum AMH concentration and US-PCOM define the same PCOS phenotype in the clinical setting is unclear in view of our current findings. When we applied an in-house specific cut-off to set normal or increased serum AMH levels as recommended [1], increased serum AMH showed a good specificity for identifying PCOM but a very poor sensitivity and negative predictive value. Therefore, increased serum AMH concentrations would be useful to ascertain PCOM but would not rule it out when a normal AMH result is present.

Earlier reports [25, 26], but not all [27], suggested a good concordance between serum AMH and US for the diagnosis of PCOM. Former studies applied standardized US protocols and state-of-the-art equipment, while our observations were conducted in the context of real-life clinical practice by observers that, not rarely, had to use US probes with a maximum frequency below 8 MHz. In addition, although our local AMH cutoff is similar to that obtained by others from population-based studies [28], previous studies set serum AMH thresholds for defining PCOM that were lower than those derived from our control population. Such threshold values were obtained from normal cycling women with US-PCOM [29], or from ROC analyses in which the diagnosis of PCOM relied on Rotterdam criteria [26]. Although whether excluding otherwise healthy women with US-PCOM from the process of establishing a normal reference range for serum AMH levels may be debatable, the fact that US was not performed in any of our controls made it impossible for us to exclude this possibility. Therefore, the inclusion of certain number of non-hyperandrogenic control women presenting with sonographic appearance of PCOM, but not meeting other PCOS criteria, may have increased our AMH normality cutoff. Either way, lowering AMH cut-offs to the level suggested by ROC analysis of patients in whom US was available would have resulted in an unacceptable low specificity. In other vein, our reference population for establishing AMH normative values included both non-obese and obese non-hyperandrogenic women with regular menses. Obesity may be negatively associated with ovarian reserve decreasing circulating AMH. In conceptual agreement, our obese control women presented with AMH levels mildly lower than those of their non-obese counterparts (data not shown). However, the impact of adiposity on AMH levels in otherwise healthy women with regular menses remains largely uncertain [30].

Nevertheless, the possibility exists that PCOM defined by US and serum AMH levels identify two subgroups of women with subtle phenotype differences [31]. Taken together, our results suggest that US and serum AMH are not completely interchangeable to diagnose PCOM, and, accordingly, it would be advisable a two-step process for PCOM phenotyping in women with a potential ovulatory or non-hyperandrogenic PCOS if US examination is not necessary to rule out other suspected condition. The initial step would consist of measuring serum AMH concentrations, giving its very good predictive value for PCOM, reserving US examination for women in whom AMH concentrations were not increased.

Our work had several weaknesses that may limit a broad generalization such as the use of two different immunoassays throughout the study period in our routine laboratory, possible technical issues with US examinations, and the absence of US examination in both our control population and some patients already discussed. Another potential factor that may have influenced our results resulted from referral bias. Earlier studies including our own suggested that patients referring to a Reproductive Endocrinology clinic may be more hyperandrogenic and more obese than those in the general population [13, 32]. Such a referral bias would explain not only the high frequency of obesity and abnormal glucose tolerance in our women who did not fulfil PCOS criteria after LC–MS/MS and serum AMH assessments, but also their presence in our population of control women that was partially composed of individuals seeking advice for weight excess. Finally, not all women with group II WHO OD in our series received an US examination despite presenting with normal AMH concentrations. Thus, we cannot rule out that PCOM was present in them, yet obesity itself may associate ovulatory dysfunction even in eumenorrheic women [33], regardless of circulating androgen levels [34].

In short, immunoassays are not accurate enough to permit a reliable diagnosis and phenotyping of PCOS, possibly the most common endocrine and metabolic disorder of women [35] with consequences that extend into the menopausal age [36]. Hence, even if integration into routine clinical practice of MS-based methods for sex-steroid measurements might be considered unrealistic by some, arguing complexity issues and costs, the vast numbers of women with PCOS worldwide deserve a reliable method for diagnosis, facilitating attending physicians with the correct tools for clinical decision-making. Our data cast some doubt upon the interchangeability of serum AMH and US for the diagnosis of PCOM in routine clinical practice, mostly because of the low negative predictive value of the former. Nonetheless, increased serum AMH measurements in those women without features of classic PCOS would avoid about 30% of US examinations in a first step.

## Supplementary Information

Below is the link to the electronic supplementary material.

Supplementary file1 (DOCX 635 KB)

## Data Availability

All data sets generated and/or analyzed during the current study are not publicly available but are available from the corresponding author on reasonable request.

## References

[1] Teede HJ, Tay CT, Laven JJE et al (2023) Recommendations from the 2023 international evidence-based guideline for the assessment and management of polycystic ovary syndrome. Eur J Endocrinol 189:G43–G64. 10.1093/ejendo/lvad096 37580861 10.1093/ejendo/lvad096

[2] Büttler RM, Kruit A, Blankenstein MA, Heijboer AC (2013) Measurement of dehydroepiandrosterone sulphate (DHEAS): a comparison of Isotope-Dilution Liquid Chromatography Tandem Mass Spectrometry (ID-LC-MS/MS) and seven currently available immunoassays. Clin Chim Acta 424:22–26. 10.1016/j.cca.2013.04.028 23665079 10.1016/j.cca.2013.04.028

[3] Tosi F, Fiers T, Kaufman JM et al (2016) Implications of androgen assay accuracy in the phenotyping of women with polycystic ovary syndrome. J Clin Endocrinol Metab 101:610–618. 10.1210/jc.2015-2807 26695861 10.1210/jc.2015-2807

[4] Pasquali R, Zanotti L, Fanelli F et al (2016) Defining hyperandrogenism in women with polycystic ovary syndrome: a challenging perspective. J Clin Endocrinol Metab 101:2013–2022. 10.1210/jc.2015-4009 26964728 10.1210/jc.2015-4009

[5] Ankarberg-Lindgren C, Becker C, Svala E, Ryberg H (2023) Methodological considerations in determining sex steroids in children: comparison of conventional immunoassays with liquid chromatography-tandem mass spectrometry. Clin Chem Lab Med 7:2023–2344. 10.1515/cclm-2023-0344 10.1515/cclm-2023-0344 37540832

[6] Rosner W, Auchus RJ, Azziz R, Sluss PM, Raff H (2007) Position statement: utility, limitations, and pitfalls in measuring testosterone: an Endocrine Society position statement. J Clin Endocrinol Metab 92:405–413. 10.1210/jc.2006-1864 17090633 10.1210/jc.2006-1864

[7] Casals G, Costa RF, Rull EU, Escobar-Morreale HF, Argente J, Sesmilo G, Biagetti B (2023) Recommendations for the measurement of sexual steroids in clinical practice. A position statement of SEQC(ML)/SEEN/SEEP. Adv Lab Med 4:52–69. 10.1515/almed-2023-0020 37359897 10.1515/almed-2023-0020 PMC10197192

[8] Moghetti P, Tosi F, Bonin C et al (2013) Divergences in insulin resistance between the different phenotypes of the polycystic ovary syndrome. J Clin Endocrinol Metab 98:E628–E637. 10.1210/jc.2012-3908 23476073 10.1210/jc.2012-3908

[9] Copp T, Doust J, McCaffery K, Hersch J, Jansen J (2021) Polycystic ovary syndrome: why widening the diagnostic criteria may be harming women. BMJ 373:n700. 10.1136/bmj.n700 33863701 10.1136/bmj.n700

[10] Dewailly D, Lujan ME, Carmina E, Cedars MI, Laven J, Norman RJ, Escobar-Morreale HF (2014) Definition and significance of polycystic ovarian morphology: a task force report from the Androgen Excess and Polycystic Ovary Syndrome Society. Hum Reprod Update 20:334–352. 10.1093/humupd/dmt061 24345633 10.1093/humupd/dmt061

[11] Teede H, Misso M, Tassone EC et al (2019) Anti-Müllerian hormone in PCOS: a review informing international guidelines. Trends Endocrinol Metab 30:467–478. 10.1016/j.tem.2019.04.006 31160167 10.1016/j.tem.2019.04.006

[12] Escobar-Morreale HF (2018) Polycystic ovary syndrome: definition, aetiology, diagnosis and treatment. Nat Rev Endocrinol 14:270–284. 10.1038/nrendo.2018.24 29569621 10.1038/nrendo.2018.24

[13] Luque-Ramirez M, Alpanes M, Sanchon R, Fernandez-Duran E, Ortiz-Flores AE, Escobar-Morreale HF (2015) Referral bias in female functional hyperandrogenism and polycystic ovary syndrome. Eur J Endocrinol 173:603–610. 10.1530/EJE-15-0646 26243032 10.1530/EJE-15-0646

[14] Luque-Ramírez M, Jiménez-Mendiguchia L, García-Cano A et al (2018) Certified testosterone immunoassays for hyperandrogenaemia. Eur J Clin Invest 48:e13029. 10.1111/eci.13029 30229887 10.1111/eci.13029

[15] Rotterdam ESHRE/ASRM-Sponsored PCOS Consensus Workshop Group (2004) Revised 2003 consensus on diagnostic criteria and long-term health risks related to polycystic ovary syndrome. Fertil Steril 81:19–25. 10.1016/j.fertnstert.2003.10.004 10.1016/j.fertnstert.2003.10.004 14711538

[16] Ostchega Y, Seu R, Sarafrazi N, Zhang G, Hughes JP, Miller I (2019) Waist Circumference Measurement Methodology Study: National Health and Nutrition Examination Survey, 2016. Vital Health Stat 2(2):1–20 30707674

[17] Matsuda M, DeFronzo RA (1999) Insulin sensitivity indices obtained from oral glucose tolerance testing: comparison with the euglycemic insulin clamp. Diabetes Care 22:1462–1470. 10.2337/diacare.22.9.1462 10480510 10.2337/diacare.22.9.1462

[18] ElSayed NA, Aleppo G, Aroda VR et al (2023) Classification and Diagnosis of Diabetes: Standards of Care in Diabetes-2023. Diabetes Care. 10.2337/dc23-S002 36507649 10.2337/dc23-S002 PMC9810477

[19] Dietz de Loos A, Hund M, Buck K, Meun C, Sillman J, Laven JSE (2021) Antimüllerian hormone to determine polycystic ovarian morphology. Fertil Steril 116:1149–1157. 10.1016/j.fertnstert.2021.05.094 34579824 10.1016/j.fertnstert.2021.05.094

[20] Cao ZT, Botelho JC, Rej R, Vesper H, Astles JR (2019) Impact of testosterone assay standardization efforts assessed via accuracy-based proficiency testing. Clin Biochem 68:37–43. 10.1016/j.clinbiochem.2019.03.014 30928392 10.1016/j.clinbiochem.2019.03.014

[21] Livingston M, Downie P, Hackett G, Marrington R, Heald A, Ramachandran S (2020) An audit of the measurement and reporting of male testosterone levels in UK clinical biochemistry laboratories. Int J Clin Pract 74:e13607. 10.1016/10.1111/ijcp.13607 32649008 10.1111/ijcp.13607

[22] Rosner W, Vesper H (2010) Toward excellence in testosterone testing: a consensus statement. J Clin Endocrinol Metab 95:4542–4548. 10.1210/jc.2010-1314 20926540 10.1210/jc.2010-1314

[23] Azziz R, Carmina E, Dewailly D et al (2009) The Androgen Excess and PCOS Society criteria for the polycystic ovary syndrome: the complete task force report. Fertil Steril 91:456–488. 10.1016/j.fertnstert.2008.06.035 18950759 10.1016/j.fertnstert.2008.06.035

[24] Yuwen T, Yang Z, Cai G, Feng G, Liu Q, Fu H (2023) Association between serum AMH levels and IVF/ICSI outcomes in patients with polycystic ovary syndrome: a systematic review and meta-analysis. Reprod Biol Endocrinol 21:95. 10.1186/s12958-023-01153-y 37872575 10.1186/s12958-023-01153-y PMC10591359

[25] Lie Fong S, Laven JSE, Duhamel A, Dewailly D (2017) Polycystic ovarian morphology and the diagnosis of polycystic ovary syndrome: redefining threshold levels for follicle count and serum anti-Müllerian hormone using cluster analysis. Hum Reprod 32:1723–1731. 10.1093/humrep/dex226 28854584 10.1093/humrep/dex226

[26] Lauritsen MP, Bentzen JG, Pinborg A et al (2014) The prevalence of polycystic ovary syndrome in a normal population according to the Rotterdam criteria versus revised criteria including anti-Mullerian hormone. Hum Reprod 29:791–801. 10.1093/humrep/det469 24435776 10.1093/humrep/det469

[27] Carmina E, Campagna AM, Fruzzetti F, Lobo RA (2016) AMH measurement versus ovarian ultrasound in the diagnosis of polycystic ovary syndrome in different phenotypes. Endocr Pract 22:287–293. 10.4158/EP15903.OR 26523627 10.4158/EP15903.OR

[28] Piltonen TT, Komsi E, Morin-Papunen LC et al (2023) AMH as part of the diagnostic PCOS workup in large epidemiological studies. Eur J Endocrinol 188:547–554. 10.1093/ejendo/lvad065 37294941 10.1093/ejendo/lvad065

[29] Dewailly D, Gronier H, Poncelet E et al (2011) Diagnosis of polycystic ovary syndrome (PCOS): revisiting the threshold values of follicle count on ultrasound and of the serum AMH level for the definition of polycystic ovaries. Hum Reprod 26:3123–3129. 10.1093/humrep/der297 21926054 10.1093/humrep/der297

[30] Nguyen MT, Krishnan S, Phatak SV, Karakas SE (2023) Anti-Müllerian hormone-based phenotyping identifies subgroups of women with polycystic ovary syndrome with differing clinical and biochemical characteristics. Diagnostics (Basel) 13:500. 10.3390/diagnostics13030500 36766605 10.3390/diagnostics13030500 PMC9914382

[31] Oldfield AL, Kazemi M, Lujan ME (2021) Impact of obesity on anti-Mullerian hormone (AMH) levels in women of reproductive age. J Clin Med 10:3192. 10.3390/jcm10143192 34300357 10.3390/jcm10143192 PMC8306853

[32] Ezeh U, Yildiz BO, Azziz R (2013) Referral bias in defining the phenotype and prevalence of obesity in polycystic ovary syndrome. J Clin Endocrinol Metab 98:E1088–E1096. 10.1210/jc.2013-1295 23539721 10.1210/jc.2013-1295 PMC3667270

[33] Oldfield AL, Vanden Brink H, Carter FE, Jarrett BY, Lujan ME (2023) Obesity is associated with alterations in antral follicle dynamics in eumenorrheic women. Hum Reprod 38:459–470. 10.1093/humrep/dead007 36708012 10.1093/humrep/dead007 PMC9977134

[34] Bloom MS, Perkins NJ, Sjaarda LA et al (2021) Adiposity is associated with anovulation independent of serum free testosterone: A prospective cohort study. Paediatr Perinat Epidemiol 35:174–183. 10.1111/ppe.12726 33107110 10.1111/ppe.12726 PMC7878298

[35] Carmina E, Lobo RA (1999) Polycystic ovary syndrome (PCOS): arguably the most common endocrinopathy is associated with significant morbidity in women. J Clin Endocrinol Metab 84:1897–1899. 10.1210/jcem.84.6.5803 10372683 10.1210/jcem.84.6.5803

[36] Millán-de-Meer M, Luque-Ramírez M, Nattero-Chávez L, Escobar-Morreale HF (2023) PCOS during the menopausal transition and after menopause: a systematic review and meta-analysis. Hum Reprod Update 29:741–772. 10.1093/humupd/dmad015 37353908 10.1093/humupd/dmad015

