# Supplementary material for: Influence of state-of-the-art laboratory techniques on the phenotyping of women with polycystic ovary syndrome in the clinical setting
Source: J Endocrinol Invest. 2024 Jun 24;48(1):131–43. doi: 10.1007/s40618-024-02416-0 (PMC11729125; doi:10.1007/s40618-024-02416-0)
Supplement: Supplementary file 1 — Supplementary file1 (DOCX 635 KB) [file 40618_2024_2416_MOESM1_ESM.docx]

**Supplementary Information**

Assays………………………………………………………………………………… 2

Supplementary Table S1……………………………………………………………… 4

Supplementary Table S2……………………………………………………………… 5

Supplementary Table S3……………………………………………………………… 6

Supplementary Figure S1……………………………………………………………... 7

Supplementary Figure S2…………………………………………………………….... 8

Supplementary Figure S3……………………………………………………………… 9

References……………………………………………………………………………... 10

**Assays**

Up to February 2020, routine serum samples were assayed in duplicate for total testosterone (T) using a direct unextracted RIA (Spectria® Testosterone RIA, Orion Diagnostica Oy, Espoo, Finland) with analytical and functional sensitivity of < 0.1 and 0.3 nM (2.9 and 8.7 ng/dL), respectively. Intra-assay coefficient of variation (CV) was < 9% for concentrations ≥ 1.3 nM (37.5 ng/dL) and inter-assay CV was 11.6% at 3.3 nM (95 ng/dL), with < 2.7% cross-reactivity for all endogenous steroids. This assay was validated against several LC-MS/MS assays using samples from healthy prepuberal and puberal girls and boys [1]. Since 2020, routine serum samples were assayed for total T in uniplicate using an automated ICLA [ADVIA Centaur ® Testosterone II Assay (TSTII), Siemens Healthcare Diagnostics Ltd., Frimley, Camberley, United Kingdom]. This immunoassay passed successfully the Centers for Diseases Control and Prevention Hormone Laboratory/Manufacturer Standardization Program (CDC HoSt) (<https://www.cdc.gov/labstandards/csp/hs_standardization.html>) from August 2016 to date. The limits of detection and quantification of this method were 0.17 and 0.24 nM (4.9 and 6.9 ng/dL), respectively. Intra-assay CV was 0.37 nM (10.6 ng/dL), inter-assay CV was 0.92 nM (26.7 ng/dL), and cross-reactivity was < 10%. Both methods – RIA and ICLA – have shown a moderate correlation (r: 0.673; *P* < 0.001) among them in our population [2], but the concordance between both techniques was poor-to-fair in terms of absolute agreement.

Up to 2020, routine measurements for androstenedione (A4) and dehydroepiandrosterone-sulphate (DHEAS) used an automated ICLA (IMMULITE 2000, Siemens Healthcare Sector, Erlangen, Germany) with lower limits of quantification (LLOQ) of 10.1 nM (2.9 ng/mL) for A4 and 0.4 μM (150 ng/mL) for DHEAS. The mean intra-assay and inter-assay CVs were < 10% for all these assays. From 2020 to date, our local laboratory used the Liaison XL Diasorin assay for A4 and the Advia Centaur XP Siemens assay for DHEAS. Their LLOQ were 0.84 nM (0.24 ng/mL) and 0.4 μM (150 ng/mL), respectively, and intra-assay and inter-assay CVs were < 10% for both methods.

Stored aliquots of serum samples were assayed for total T and A4 by LC–MS/MS at the Laboratory of Clinical Biology of the University of Ghent, Belgium, using an AB Sciex 6500 triple-quadrupole mass spectrometer (AB Sciex, Toronto, Canada). The LLOQ was 0.04 nM (1.2 ng/dl) for total T and the inter-assay CV was 8.3% at 1.27 nM (36.7 ng/dl) and 3.1% at 10.7 nM (307.8 ng/dl). Serum LLOQ was < 0.17 nM (0.05 ng/mL) for A4 and the inter-assay CV was < 7.1%.

We calculated free T (cFT) concentrations from total T measured by routine immunoassays and LC-MS/MS assays, sex hormone-binding globulin (SHBG) concentrations, and albumin using an equation based on the law of mass action according to Vermeulen *et al*. [3] SHBG was measured using an automated ICLA (IMMULITE 2000, Siemens Healthcare Sector, Erlangen, Germany) with a LLOQ of 0.02 nM and mean intra-assay and inter-assay CVs < 10%. In our population, cFT determined from RIA- or ICLA-derived total T concentrations have shown a high positive correlation coefficient (r: 0.829; *P* < 0.001) and a good-to-excellent consistency, although their absolute agreement was poor [2].

Serum anti-müllerian hormone (AMH) concentrations were measured using an automated ICLA on a Cobas e601^®^ analyser (Elecsys^®^, Roche Diagnostics, Germany). The assay limits of detection and LLOQ were 0.07 and 0.21 pM (0.01 and 0.03 ng/mL), respectively. The intra-assay and inter-assay CVs were < 4%. The limit above the measuring range was 164.22 pM (23 ng/mL).

## Circulating HDL-cholesterol, total cholesterol, and triglyceride levels were determined by enzymatic methods (formerly Menarini Diagnostica, and Architect C.16000 since 2013). LDL-cholesterol concentrations were estimated by the Friedewald’s equation.

**Supplementary Table S1.** Clinical features and circulating testosterone concentrations as a function of routine immunoassays.

|  | **RIA**  (n = 306) | | | **ICLA**  (n = 53) | | | *P* |
| --- | --- | --- | --- | --- | --- | --- | --- |
| *Age, years* | 26 | ± | 7 | 27 | ± | 6 | 0.892 |
| *Body mass index, kg/m^2^* | 30.0 | ± | 7.3 | 28.3 | ± | 7.2 | 0.134 |
| *Waist circumference, cm* | 82 | ± | 17 | 85 | ± | 17 | 0.302 |
| *Waist-to-hip ratio* | 0.80 | ± | 0.11 | 0.80 | ± | 0.09 | 0.530 |
| *Total testosterone, nM* | 2.5 | ± | 1.2 | 1.5 | ± | 0.7 | <0.001 |
| *Hyperandrogenemia by total testosterone* | 141 (46.1%) | | | 33 (62.3%) | | | 0.029 |
| *Calculated free testosterone, pM* | 41.5 | ± | 21.4 | 29.3 | ± | 20.9 | <0.001 |
| *Hyperandrogenemia by calculated free testosterone* | 118 (38.6%) | | | 19 (35.8%) | | | 0.707 |

Data are shown as mean ± standard deviation and raw numbers (percentage) as appropriate. Continuous and categorical variables were compared by Mann Whitney *U* tests and χ^2^ tests, respectively.

**Supplementary Table S2.** Anthropometric, clinical, and metabolic variables as a function of normal or increased concentrations of anti-müllerian hormone in women with PCOS, and in control women.

|  | **All PCOS phenotypes**  **as a whole** | | | | | | **Control**  **women** | | |
| --- | --- | --- | --- | --- | --- | --- | --- | --- | --- |
|  | **Normal AMH** | | | **↑AMH** | | |  |  |  |
|  | (n =248) | | | (n = 109) | | | (n = 91) | | |
| *Age, years* | 26 | ± | 7^*^ | 27 | ± | 6 | 29 | ± | 6 |
| *Body mass index, kg/m^2^* | 28.2 | ± | 7.6^*^ | 24.9 | ± | 6.1^*^ | 26.6 | ± | 7.6 |
| *Obesity^a^* | 93 (38%) | | | 28 (26%) | | | 29 (32%) | | |
| *Waist circumference,cm* | 83 | ± | 18 | 79 | ± | 15 | 82 | ± | 17 |
| *Waist-to-hip ratio* | 0.80 | ± | 0.11 | 0.80 | ± | 0.10 | 0.79 | ± | 0.09 |
| *Systolic blood pressure, mmHg^a^* | 120 | ± | 13^*^ | 116 | ± | 16 | 113 | ± | 12 |
| *Diastolic blood pressure, mmHg* | 76 | ± | 10^*^ | 75 | ± | 9 | 73 | ± | 10 |
| *Fasting glucose, mM* | 5.0 | ± | 0.6 | 4.9 | ± | 0.5 | 4.9 | ± | 0.5 |
| *120 min-oGTT glucose, mM* | 6.5 | ± | 2.2 | 6.4 | ± | 1.7 | 6.3 | ± | 1.5 |
| *AUC _oGTT glucose_, mM*120 min* | 246 | ± | 187 | 252 | ± | 153 | 246 | ± | 121 |
| *Insulin sensitivity index^a^* | 5.9 | ± | 4.5^*^ | 6.9 | ± | 4.3 | 7.2 | ± | 3.6 |
| *Abnormal glucose tolerance* | 67 (28%) | | | 26 (24%) | | | 18 (20%) | | |
| *Total cholesterol, mM^a^* | 4.5 | ± | 0.8 | 4.7 | ± | 0.9 | 4.6 | ± | 0.9 |
| *HDL, cholesterol, mM^a^* | 1.3 | ± | 0.3^*^ | 1.5 | ± | 0.4 | 1.5 | ± | 0.4 |
| *LDL-cholesterol, mM* | 2.7 | ± | 0.8 | 2.8 | ± | 0.7 | 2.8 | ± | 0.7 |
| *Triglycerides, mM* | 1.0 | ± | 0.5 | 1.0 | ± | 0.6 | 0.9 | ± | 0.5 |
| *Anti-müllerian hormone, pM^a^* | 30.4 | ± | 12.9^*^ | 85.7 | ± | 37.1^*^ | 22.4 | ± | 15.4 |
| *PCOM by ultrasound* | 127/154 (83%) | | | 51/55 (93%) | | | - | | |
| *Ovulatory dysfunction* | 227 (92%) | | | 106 (97%) | | | 0 (0%) | | |
| *Total testosterone, nM^a^* | 1.2 | ± | 0.5^*^ | 1.9 | ± | 0.9^*^ | 0.8 | ± | 0.3 |
| *Sex hormone-binding globulin,nM^a^* | 40 | ± | 24^*^ | 54 | ± | 30 | 54 | ± | 25 |
| *Calculated free testosterone, pM^a^* | 21.5 | ± | 9.8^*^ | 27.9 | ± | 15.7^*^ | 14.0 | ± | 5.3 |
| *Androstenedione, nM^a^* | 5.8 | ± | 2.0^*^ | 8.1 | ± | 3.1^*^ | 4.5 | ± | 1.5 |
| *Dehydroepiandrosterone-sulphate, μM* | 6.6 | ± | 3.2 | 6.0 | ± | 3.2 | 5.0 | ± | 2.4 |

Data are shown as means ± SD or counts (%). Continuous variables were compared among groups by univariate Welch’s ANOVA followed by Games-Howell’s post-hoc analysis or univariate general linear models adjusting by age and followed by Bonferroni’s post-hoc analysis. Dichotomous variables were compared by binary logistic regression analysis adjusting by age.

^*^*P* <0.05 vs. control group. ^a^*P* < 0.05 for comparisons between both patients with normal or increased AMH (as defined by values above the 95^th^ percentile of the group of control women).

**Supplementary Table S3.** Anthropometric, clinical, and metabolic variables as a function of normal or increased concentrations of anti-müllerian hormone in patients without classic PCOS phenotypes and in control women.

|  | **Patients without**  **classic PCOS phenotype** | | | | | | **Control**  **women** | | |
| --- | --- | --- | --- | --- | --- | --- | --- | --- | --- |
|  | **Normal AMH** | | | **↑AMH** | | |  |  |  |
|  | (n = 89) | | | (n = 30) | | | (n = 91) | | |
| *Age, years* | 28 | ± | 7 | 30 | ± | 5 | 29 | ± | 6 |
| *Body mass index, kg/m^2 a^* | 27.5 | ± | 6.7 | 23.2 | ± | 4.4^*^ | 26.6 | ± | 7.6 |
| *Obesity* | 33 (37%) | | | 4 (13%) | | | 29 (32%) | | |
| *Waist circumference,cm* | 82 | ± | 17 | 78 | ± | 11 | 82 | ± | 17 |
| *Waist-to-hip ratio* | 0.78 | ± | 0.07 | 0.80 | ± | 0.07 | 0.79 | ± | 0.09 |
| *Systolic blood pressure, mmHg* | 120 | ± | 13^*^ | 116 | ± | 9 | 113 | ± | 12 |
| *Diastolic blood pressure, mmHg* | 76 | ± | 10 | 74 | ± | 10 | 73 | ± | 10 |
| *Fasting glucose, mM* | 4.9 | ± | 0.4 | 4.8 | ± | 0.5 | 4.9 | ± | 0.5 |
| *120 min-oGTT glucose, mM* | 6.6 | ± | 2.6 | 5.6 | ± | 1.4 | 6.3 | ± | 1.5 |
| *AUC _oGTT glucose_, mM*120 min* | 259 | ± | 236 | 187 | ± | 144 | 246 | ± | 121 |
| *Insulin sensitivity index^a^* | 7.0 | ± | 5.4 | 9.4 | ± | 4.2^*^ | 7.2 | ± | 3.6 |
| *Abnormal glucose tolerance* | 23 (26%) | | | 3 (10%) | | | 18 (20%) | | |
| *Total cholesterol, mM* | 4.3 | ± | 0.7^*^ | 4.7 | ± | 0.8 | 4.6 | ± | 0.9 |
| *HDL, cholesterol, mM^a^* | 1.4 | ± | 0.4 | 1.6 | ± | 0.4 | 1.5 | ± | 0.4 |
| *LDL-cholesterol, mM* | 2.5 | ± | 0.6 | 2.7 | ± | 0.7 | 2.8 | ± | 0.7 |
| *Triglycerides, mM* | 0.9 | ± | 0.5 | 0.8 | ± | 0.4 | 0.9 | ± | 0.5 |
| *Anti-müllerian hormone, pM^a^* | 27.3 | ± | 12.4^*^ | 75.1 | ± | 16.1^*^ | 22.4 | ± | 15.4 |
| *PCOM by ultrasound* | 71/77 (92%) | | | 23/23 (100%) | | | - | | |
| *Ovulatory dysfunction* | 68 (76%) | | | 27 (90%) | | | 0 (0%) | | |
| *Total testosterone, nM^a^* | 1.0 | ± | 0.3 | 1.2 | ± | 0.4^*^ | 0.8 | ± | 0.3 |
| *Sex hormone-binding globulin, nM^a^* | 48 | ± | 25^*^ | 61 | ± | 24 | 54 | ± | 25 |
| *Calculated free testosterone, pM* | 15.4 | ± | 5.2 | 15.1 | ± | 6.3 | 14.0 | ± | 5.3 |
| *Androstenedione, nM^a^* | 4.8 | ± | 1.3 | 5.6 | ± | 1.9^*^ | 4.5 | ± | 1.5 |
| *Dehydroepiandrosterone-sulphate, μM^a^* | 5.7 | ± | 2.8 | 4.2 | ± | 1.7 | 5.0 | ± | 2.4 |

Data are shown as means ± SD or counts (percentage). Continuous variables were compared among groups by univariate ANOVA or Welch-ANOVA followed by Tukey’s or Games-Howell’s post-hoc analysis. Dichotomous variables were compared by binary logistic regression analysis.

^*^ *P* <0.05 vs. control group. ^a^*P* < 0.05 between both patients with normal or increased AMH (as defined by values above the 95^th^ percentile of the group of control women).

**Supplementary Figure S1** Comparisons of circulating total testosterone and androstenedione measured by routine immunoassays and by gold-standard liquid chromatography-tandem mass spectrometry (LC-MS/MS).

Panel A: Correlation plots. Solid and dashed black lines represent *β* coefficients of simple linear regression and its 95% confidence intervals. Purple and red points and lines represent RIA and IQLA total testosterone measurements, respectively. ICC: intra-class correlation. *r*: Pearson's correlation coefficient.

Panel B: Bland-Altman plots.

Panel C: Comparisons using the ratio method. Total testosterone and androstenedione concentrations measured by LC-MS/MS method are represented on the *x*-axis, and the ratio among the routine immunoassays and the LC-MS/MS method are on the *y*-axis

**
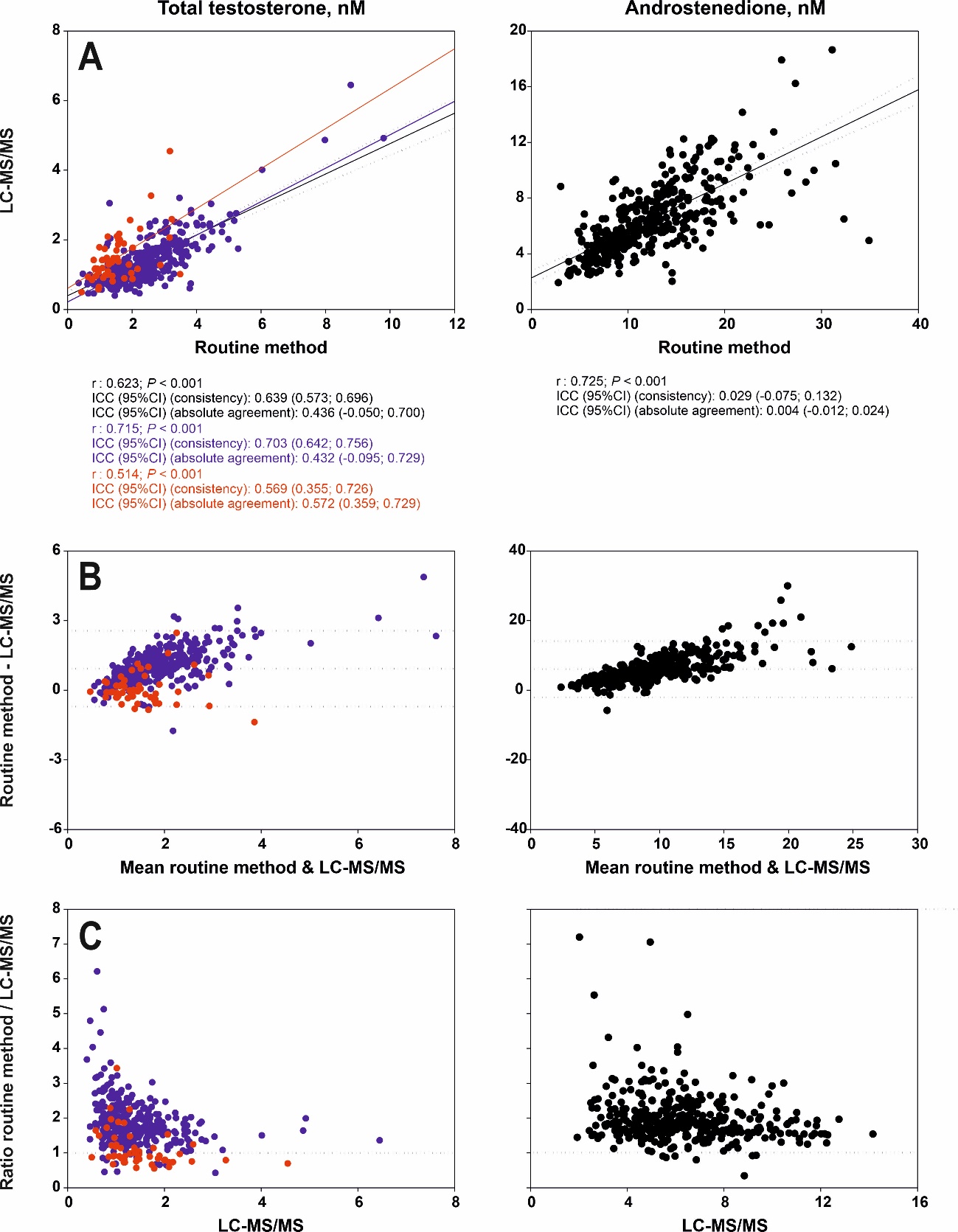
**

**Supplementary Figure S2.** Anti-müllerian hormone (AMH) performance in diagnosing ultrasound polycystic ovarian morphology (PCOM). According to the Youden’s index, the optimal AMH threshold value would be 16.3 pM (2.3 ng/mL), which showed 0.92 sensitivity for diagnosing ultrasound PCOM, but only 0.36 specificity.


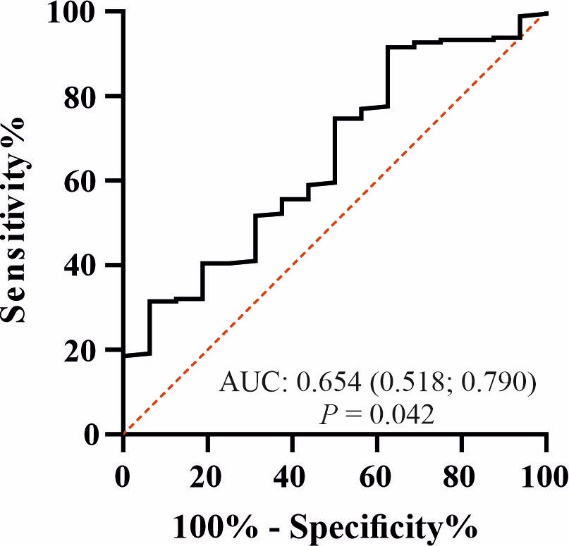


**Supplementary Figure S3.** Frequency of abnormal glucose tolerance (prediabetes or diabetes mellitus) as a function of PCOS phenotypes and compared with non-hyperandrogenic control women presenting with regular menses. Analyses used binary logistic regression analysis adjusting by age. Figures above bars show counts (percentage) of events. ^*^*P* < 0.05 vs. control women [OR: 1.86 (1.02; 3.40)].


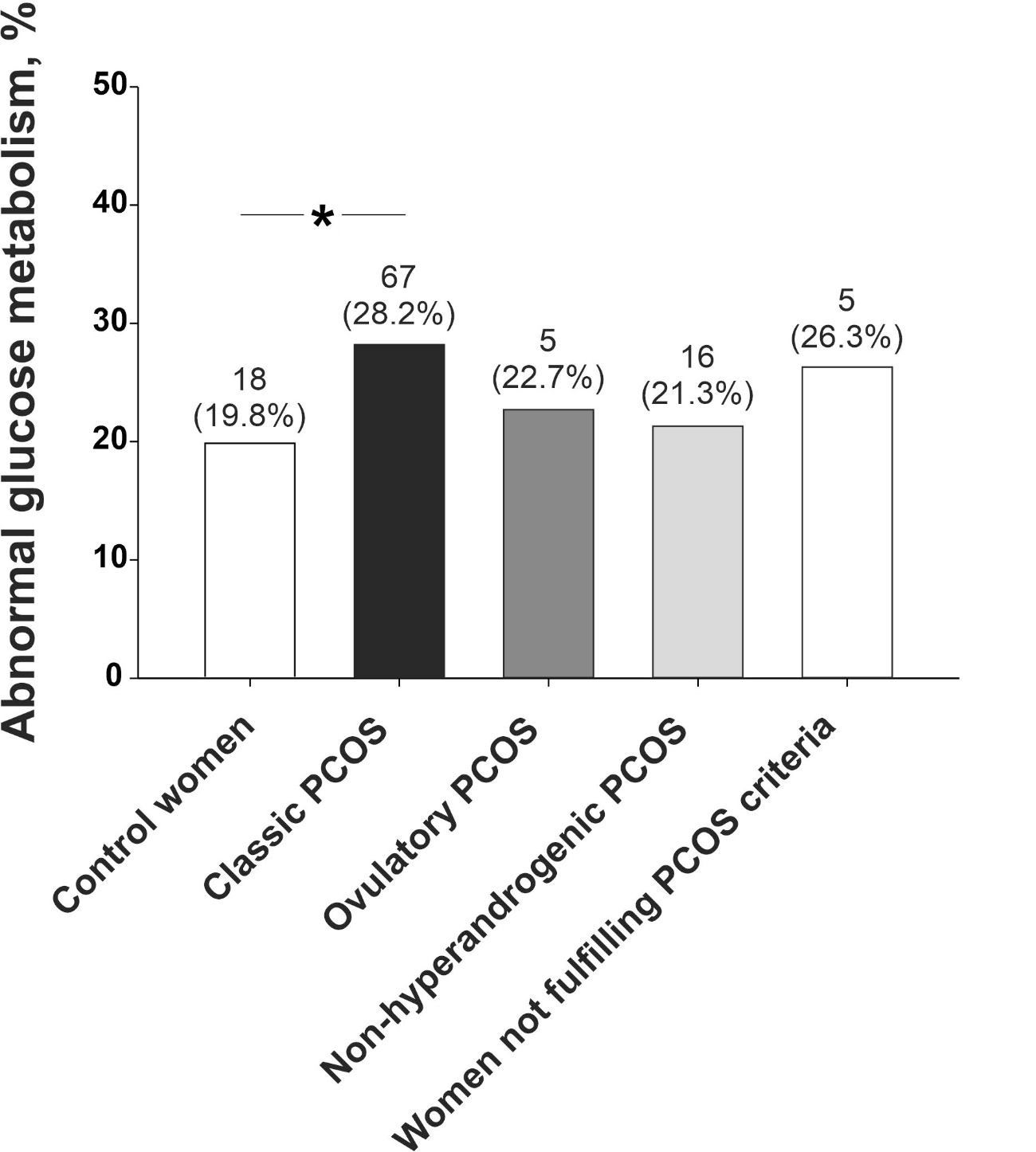


**References**

1 Ankarberg-Lindgren C, Norjavaara E. Sensitive RIA measures testosterone concentrations in prepubertal and pubertal children comparable to tandem mass spectrometry. *Scand J Clin Lab Invest* 2015; **75:** 341-4. https://doi.org/ 10.3109/00365513.2014.942694.

2 Luque-Ramírez M, Jiménez-Mendiguchia L, García-Cano A*, et al.* Certified testosterone immunoassays for hyperandrogenaemia. *Eur J Clin Invest* 2018; **48:** e13029. https://doi.org/ 10.1111/eci.13029.

3 Vermeulen A, Verdonck L, Kaufman JM. A critical evaluation of simple methods for the estimation of free testosterone in serum. *J Clin Endocrinol Metab* 1999; **84:** 3666-72. https://doi.org/ 10.1210/jcem.84.10.6079.
